# Diagnosis of Biliary Strictures Using Probe-Based Confocal Laser Endomicroscopy under the Direct View of Peroral Cholangioscopy: Results of a Prospective Study (with Video)

**DOI:** 10.1155/2020/6342439

**Published:** 2020-12-31

**Authors:** Yuki Tanisaka, Shomei Ryozawa, Kouichi Nonaka, Masami Yasuda, Akashi Fujita, Tomoya Ogawa, Masafumi Mizuide, Tomoaki Tashima, Ryuichiro Araki

**Affiliations:** ^1^Department of Gastroenterology, Saitama Medical University International Medical Center, Japan; ^2^Department of Digestive Endoscopy, Tokyo Women's Medical University, Japan; ^3^Department of Pathology, Saitama Medical University International Medical Center, Japan; ^4^Saitama Medical University, Community Health Science Center, Japan

## Abstract

**Background:**

The accurate diagnosis of biliary strictures remains problematic. The aim of the present study was to prospectively evaluate the clinical utility of probe-based confocal laser endomicroscopy (pCLE) under the direct view of peroral cholangioscopy (POCS) for the diagnosis of biliary strictures.

**Methods:**

Consecutive patients with biliary strictures were included. We investigated sensitivity, specificity, and accuracy to diagnose malignancy for (1) ERCP alone, (2) POCS, (3) pCLE under the direct view of POCS, and (4) tissue sampling under the direct view of POCS.

**Results:**

A total of 30 patients (17 with malignant lesions) were prospectively enrolled. (1) ERCP alone showed 88.2% sensitivity, 46.2% specificity, and 70% (95% confidence interval (CI), 52.1%–83.3%) accuracy. (2) POCS showed 100% sensitivity, 76.9% specificity, and 90% (95% CI, 74.4%–96.5%) accuracy. (3) pCLE under the direct view of POCS showed 94.1% sensitivity, 92.3% specificity, and 93.3% (95% CI, 78.7%–98.8%) accuracy. (4) Tissue sampling under the direct view of POCS showed 82.4% sensitivity, 100% specificity, and 90% (95% CI, 74.4%–96.5%) accuracy.

**Conclusions:**

pCLE under the direct view of POCS provided highly accurate and sensitive characterization of biliary strictures and showed the potential for more diagnostic reliability and reduction of delays in diagnosis. This trial was registered at UMIN (registration number: UMIN000033801).

## 1. Introduction

The diagnosis of biliary strictures is sometimes difficult. Although forceps biopsy under fluoroscopic guidance with endoscopic retrograde cholangiopancreatography (ERCP) is the gold standard, the diagnostic accuracy is not very high [1, 2]. Probe-based confocal laser endomicroscopy (pCLE; CholangioFlex, Cellvizio; Mauna Kea Technologies, Paris, France) has been cited in the recent American Society for Gastrointestinal Endoscopy guidelines on the management of biliary neoplasia as a useful alternative to the existing diagnostic work-up [3]. Using this method, real-time microscopic images of the bile duct tissue are generated using a dedicated confocal miniprobe that enables in vivo histological assessment in real time, known as “virtual biopsy” [4, 5].

The Miami classification consists of criteria for differentiating a healthy bile duct from a malignant stricture [6]. A series of studies evaluating the accuracy of a pCLE for diagnosing biliary strictures using the Miami classification have been published, highlighting its high sensitivity and specificity [7–13]. However, these procedures are usually performed under fluoroscopic guidance through a catheter during ERCP. Therefore, it may be doubtful whether the confocal miniprobe is properly applied to the site of interest. More recently, digital single-operator peroral cholangioscopes (POCS) (SPY-DS, SpyGlass DS; Boston Scientific Corp., Marlborough, MA, USA) have become available. The SPY-DS provides favorable visualization owing to a digital field of view of 120° and newly added injection and suction functions. Several authors have reported their clinical experience of this novel device [14–16]. It is possible to apply the confocal miniprobe accurately to the site of interest using POCS. Moreover, the pCLE findings under the direct view of POCS can be accurately matched up with biopsy tissue. These results could show more diagnostic reliability. Therefore, the aim of the present study was to prospectively evaluate the clinical utility of pCLE under the direct view of POCS for the diagnosis of biliary strictures.

## 2. Methods

### 2.1. Study Setting

This was a prospective, single-center study that was approved by the Institutional Review Board at Saitama Medical University International Medical Center (17–107). This trial was registered at UMIN (registration number: UMIN000033801). All patients provided written informed consent before undergoing the procedure.

pCLE was carried out under the direct view of POCS in consecutive patients with biliary strictures between September 2018 and March 2019. All patients underwent imaging examination, such as computed tomography (CT), magnetic resonance imaging (MRI) (MRCP), or endoscopic ultrasound (EUS) and were found to have biliary disorders.

### 2.2. Procedure Using pCLE under the Direct View of POCS

A duodenoscope (JF260V or TJF260V; Olympus Optical, Tokyo, Japan) was advanced to the ampulla, and an ERCP catheter (MTW Endoskopie, Dusseldorf, Germany) was inserted into the biliary tract. Next, a 0.025-inch guidewire (EndoSelector; Boston Scientific Corp.) was placed in the bile duct. After cholangiography was obtained using contrast medium, endoscopic sphincterotomy was carried out, if necessary. Then, SPY-DS was inserted into the bile duct under guidewire guidance. Using normal saline injection, the bile duct was observed, including the biliary stricture.

**Figure 1:**
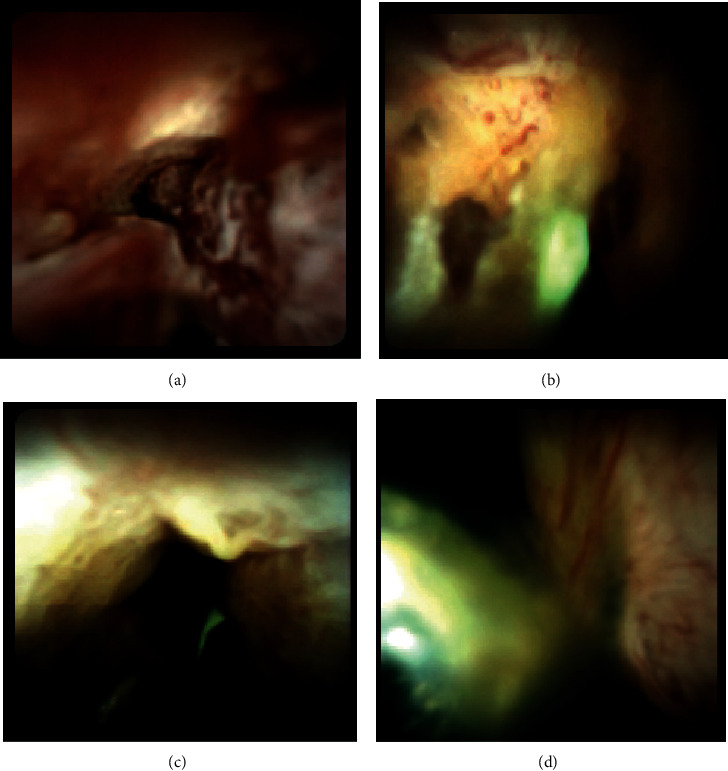
Visual findings of cholangioscopy: (a) irregular papillogranular surface, suggesting malignancy; (b) irregular thick tortuous vessels, suggesting malignancy; (c) lower homogeneous papillogranular surface without primary masses, suggesting a benign lesion; (d) fine network of thin vessels, suggesting a benign lesion.

Cholangioscopic findings were defined as either malignant or benign according to previous reports (Figure 1) [17–20]. Malignant findings included the following: (i) irregular thick tortuous vessels, (ii) easy bleeding, (iii) irregular papillogranular surface, and (iv) a nodular elevated surface such as a submucosal tumor. Benign findings included the following: (i) fine network of thin vessels and flat surface with or without shallow pseudodiverticula; (ii) lower homogeneous papillogranular surface without primary masses, suggesting hyperplasia; (iii) bumpy surface with or without pseudodiverticula, suggesting inflammation; and (iv) white surface with convergence of folds, suggesting scarring.

After observation of cholangioscopy, the guidewire was removed. Subsequently, pCLE was performed under the direct view of POCS. The CholangioFlex pCLE probe (Mauna Kea Technologies) was designed to obtain in vivo, real-time, microscopic images of the bile duct during ERCP procedures. The probe has a diameter of 0.94 mm, a field of view of 325 *μ*m, and a lateral resolution of 3.5 *μ*m. Each probe provides images from 40 to 70 *μ*m below the tissue surface. pCLE was carried out immediately after dripping three or four drops of fluorescein sodium (Alcon Laboratories Inc., Fort Worth, TX, USA) onto the lesion. The additional time for the dripping of fluorescein sodium was about 30 seconds. Thus, there is almost no additional time compared to that for intravenous injection of fluorescein sodium. Afterwards, the confocal probe was advanced through the working channel of the POCS and gently applied to the bile duct strictures to carry out confocal imaging with 12 frames per second. Intraductal images were recorded and saved to the computer connected to the probe.

**Figure 2:**
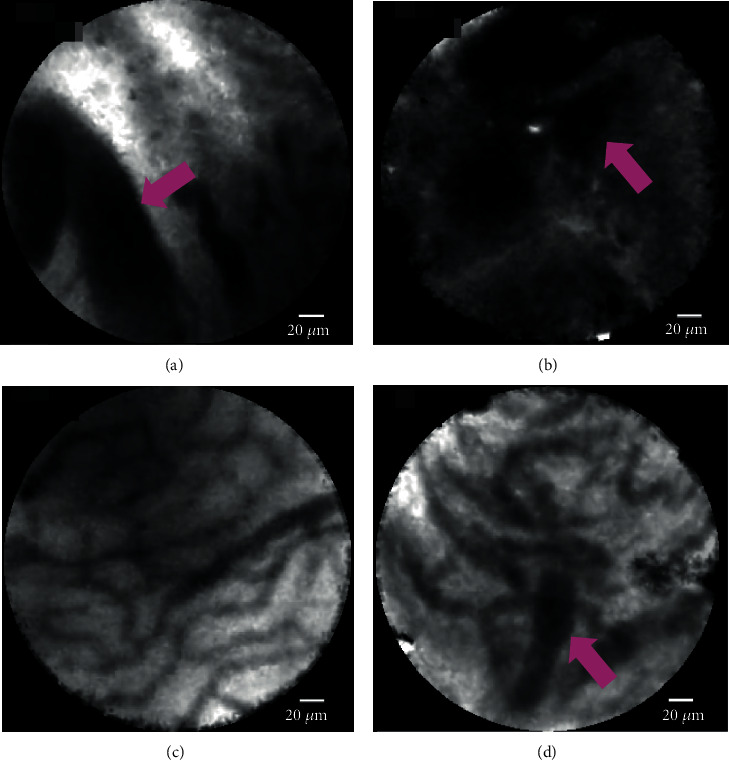
The probe-based confocal laser endomicroscopy images for biliary strictures: (a) thick dark bands (>40 *μ*m) (pink arrow) in the Miami classification; (b) dark clumps (pink arrow) in the Miami classification; (c) reticular network of thin dark branching bands (<20 *μ*m) in the Miami classification; (d) thickened reticular structure in the Paris classification (autoimmune cholangitis).

pCLE images were interpreted prospectively using the Miami classification (Figures 2(a)–2(c)), both in vivo and in real time. The criteria for diagnosis of malignancy are listed as follows:

1. Thick white bands (>20 *μ*m)
2. Thick dark bands (>40 *μ*m)
3. Dark clumps
4. Epithelium

The criteria for diagnosis of benign lesions are listed as follows:

1. Reticular network of thin dark branching bands (<20 *μ*m)
2. Light gray background
3. Blood vessels (<20 *μ*m)

After pCLE under the direct view of POCS, tissue sampling was carried out under the direct view of POCS. A forceps biopsy was taken using a SpyBite (Boston Scientific Corp.). The specimen of biliary stricture was taken at least 3 times until we confirmed sufficient materials. Tissue sampling under fluoroscopy and other cytology such as brush cytology were not performed in this study.

Two experienced endoscopists (Y.T. and S.R.) performed all procedures (ERCP, POCS, pCLE under the direct view of POCS, and tissue sampling under the direct view of POCS). They recorded a presumptive diagnosis (whether malignancy or benign) in each modality (ERCP, POCS, and pCLE under the direct view of POCS) during the procedure. Moreover, after the confirmation of tissue sampling under the direct view of POCS, they decided the further presumptive diagnosis and patient management. Biopsy specimens were examined by an experienced pathologist (M.Y.). Later, 2 investigators (K.N. and M.M.) who had previously been trained in pCLE image interpretation by using the Miami classifications and were not involved in procedures in real time were asked to review for each case and recorded the presumptive diagnosis (whether malignancy or benign) in each modality. They were blinded to the final diagnosis. The final diagnosis was made after the confirmation of the pathological findings or clinical course.

### 2.3. Definitions and Outcome Measurement

We investigated the diagnostic parameters to diagnose malignancy associated with pCLE under the direct view of POCS, including sensitivity, specificity, negative predictive value (NPV), positive predictive value (PPV), and accuracy using 2 investigators' (K.N. and M.M.) diagnosis. We investigated these parameters during ERCP procedures for (1) ERCP alone, (2) POCS, (3) pCLE under the direct view of POCS, and (4) tissue sampling under the direct view of POCS. A final diagnosis of malignancy was defined as suspicion of malignancy and malignancy on pathological findings obtained using any tissue sampling method. The final diagnosis of a benign lesion was defined if all tissue sampling methods produced a negative result, no tumor was evident on imaging, and the patient displayed a benign clinical course after a minimum of 12 months of follow-up.

We also evaluated other outcomes of pCLE under the direct view of POCS. These included the pCLE success rate under the direct view of POCS, median whole procedure time, median procedural time for pCLE under the direct view of POCS, accurate diagnostic rates of the common, hilar, or intrahepatic bile duct, and adverse events. In the clinical practice, we believe it is more difficult to observe and perform biopsy using POCS in the common bile duct, especially near the ampulla. Therefore, we considered that it would be difficult to perform pCLE under the direct view of POCS in the common bile duct. Thus, we evaluated the accurate diagnosis rate of the common bile duct and hilar or intrahepatic duct by pCLE under the direct view of POCS. Successful pCLE under the direct view of POCS was defined as the successful observation of the target area from the distal bile duct to the intrahepatic bile duct. The procedural time for pCLE under the direct view of POCS was defined as the time from the start of the insertion of the POCS to its removal after completing tissue sampling. Adverse events were assessed in accordance with the American Society for Gastrointestinal Endoscopy severity grading system [21].

### 2.4. Statistical Analysis

Continuous variables were expressed as median values. To evaluate the diagnostic yields of the proposed diagnostic methods, sensitivity, specificity, PPV, NPV, and accuracy were calculated. Two-group (POCS vs. pCLE under the direct view of POCS and pCLE under the direct view of POCS vs. tissue sampling under the direct view of POCS) comparisons of sensitivity, specificity, and accuracy were analyzed using Fisher's exact probability test. *P* values < 0.05 were considered statistically significant. All analyses were carried out using SAS JMP (version 14.1.0) and SAS (version 9.4; SAS Institute Inc., Cary, NC, USA).

## 3. Results

### 3.1. Patients

**Figure 3:**
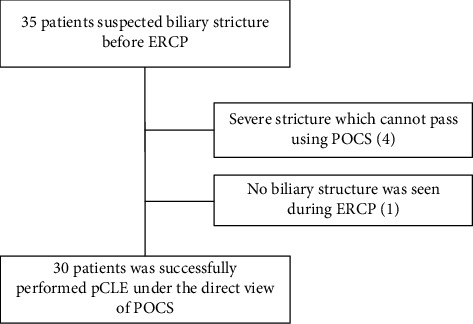
Flow chart of patient selection.

A total of 35 consecutive patients were prospectively enrolled in the present study. Among those, biliary stricture was not found on ERCP in one patient, and pCLE under the direct view of POCS was not completed due to strict biliary strictures, which did not allow POCS to pass in four patients (Figure 3). Therefore, the evaluable population consisted of 30 patients. Among 30 cases, 13 cases were indeterminate biliary strictures even though CT or MRI was performed in advance. Prior biliary stent placement was performed in 3 (10%) cases. Surgery was done in 13 (43.3%) cases. 17 (56.7%) cases were later proven to have a malignancy (16 cholangiocarcinomas and one pancreatic cancer), and 13 (43.3%) cases had benign lesions (four with autoimmune pancreatitis, two with IgG4-related sclerosing cholangitis, two with stenosis after cholecystectomy, three with chronic cholangitis, one with adenomyosis, and two with peribiliary cysts). Patient characteristics are shown in Table 1.

### 3.2. Primary Outcomes: Diagnostic Yield of pCLE under the Direct View of POCS to Diagnose Malignancy

The diagnostic yield to diagnose malignancy is shown as follows.

**Figure 4:**
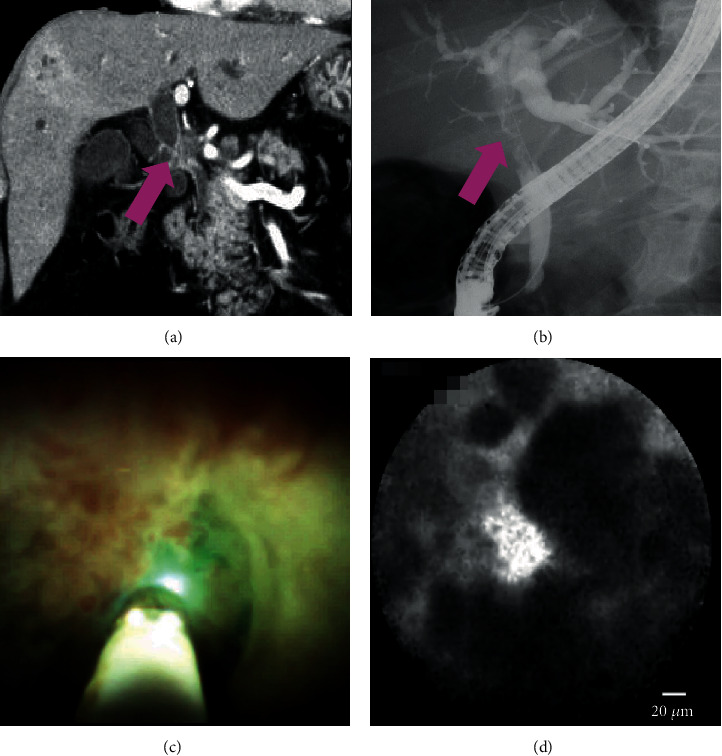
A case of malignancy: (a, b) computed tomography and cholangiography showing the biliary stricture in the hilar bile duct (pink arrow); (c) cholangioscopy showing an irregular papillogranular surface; (d) pCLE under the direct view of POCS showing dark glandular structures with irregular margins, suggestive of malignancy. Finally, the histological examination demonstrates adenocarcinoma.

(1) ERCP alone showed 88.2% sensitivity, 46.2% specificity, 75% NPV, 68.2% PPV, and 70% (95% confidence interval (CI), 52.1%–83.3%) accuracy. (2) POCS showed 100% sensitivity, 76.9% specificity, 100% NPV, 85% PPV, and 90% (95% CI, 74.4%–96.5%) accuracy. (3) pCLE under the direct view of POCS showed 94.1% sensitivity, 92.3% specificity, 92.3% NPV, 94.1% PPV, and 93.3% (95% CI, 78.7%–98.8%) accuracy. (4) Tissue sampling under the direct view of POCS showed 82.4% sensitivity, 100% specificity, 81.3% NPV, 100% PPV, and 90% (95% CI, 74.4%–96.5%) accuracy. There were no significant differences of sensitivity, specificity, and accuracy between (2) POCS and (3) pCLE under the direct view of POCS (*P* = 0.50, 0.59, and >0.99). There were also no significant differences between (3) pCLE under the direct view of POCS and (4) tissue sampling under the direct view of POCS (*P* = 0.60, >0.99, and >0.99). The results are presented in Table 2. 17 cases had malignancy in this study. Among 17 cases, we could diagnose malignancy using pCLE in 16, among which “thick dark bands” were seen in 12, “dark clumps” in 10, and both in 6. The malignant case which has undergone pCLE under the direct view of POCS is shown in Figure 4 and in Video S1.

#### 3.2.1. Other Outcomes: pCLE under the Direct View of POCS

The POCS could not pass the biliary stricture in four patients, and we could not complete successful observation of the target area. Therefore, the success rate of pCLE under the direct view of POCS was 88.2% (30/34). The median whole procedure time was 70 min (interquartile range (IQR), 60.3–79.8 min). The median procedural time for pCLE under the direct view of POCS was 42 min (IQR, 31.3–48 min). The accurate diagnosis rate of the common bile duct and the accurate diagnosis rate of the hilar or intrahepatic bile duct were 86.7% (13/15) and 100% (15/15), respectively. There was no significant difference of the accurate diagnosis rate among stricture locations (*P* = 0.48). Adverse events occurred in three patients (one with mild pancreatitis and two with mild cholangitis). These outcomes are shown in Table 3.

## 4. Discussion

The diagnosis of biliary strictures is based on a complex multimodality approach using CT, MRI (MRCP), EUS, and ERCP. Despite the advancements in imaging modalities, diagnosis of biliary strictures remains challenging in some cases. ERCP is the current gold standard for diagnosis. However, the pathological diagnosis via ERCP is limited due to the small specimen. Therefore, a novel device for the diagnosis of biliary stricture has been long-awaited. Although high sensitivity and specificity have been reported for pCLE for the diagnosis of biliary strictures using the Miami classification, it remains uncertain whether confocal miniprobes can be properly applied to the target site with the use of pCLE under fluoroscopic guidance. Therefore, we prospectively evaluated the clinical utility of pCLE under the direct view of POCS in all patients.

pCLE was performed easily and safely because we could carry out pCLE under the direct view in all patients. Biliary stricture findings by POCS and pCLE findings were matched reliably. Our results for pCLE under the direct view of POCS are comparable to those of previous studies [7–13], with an accuracy of 82–86%. Most of the previous reports have investigated pCLE under fluoroscopic guidance with ERCP. Using pCLE under fluoroscopic guidance with ERCP, it is sometimes difficult to apply the confocal miniprobe accurately to the stricture part depending on the location. Therefore, pCLE findings may be misinterpreted. The fact that POCS enables angle adjustment of the cholangioscope itself inside the bile duct and was able to properly apply the miniprobe to the site of interest using the POCS, which was highly effective for pCLE, may have contributed to our high accuracy (93.3%). Moreover, POCS is considered to have the reproducibility. The accuracy of pCLE under the direct view of POCS for 13 cases of indeterminate biliary strictures was 92.3%. In contrast, 17 cases of determinate biliary strictures in advance were 94.1% accurate. There was a similar accuracy in this study. In the previous report [22], 53 cases of pCLE under the direct view of POCS and 37 cases of pCLE under fluoroscopy were compared. The accuracy of pCLE under the direct view of POCS and pCLE under fluoroscopy was 87% and 73%, respectively. pCLE under the direct view of POCS had higher performances. This report indicates the superiority of pCLE under the direct view of POCS. Moreover, although only nonblinded investigators (Y.T., S.R.) who performed the ERCP procedure including pCLE determined further presumptive diagnosis using ERCP+POCS+pCLE under the direct view of POCS+tissue sampling, it showed 100% sensitivity, 84.6% specificity, 100% NPV, 89.5% PPV, and 93.3% accuracy. It was almost the same as pCLE under the direct view of POCS diagnosed by blinded investigators.

Although the sensitivity and accuracy of POCS were high, the specificity was relatively low. Recently, POCS has been available with a novel digital single-operator cholangioscope. This scope may have clinical benefits because of its good visibility and ease of operation. However, the false-positive rate was 23.1% (3/13) in this study. This may have been due to previous stimulation during guidewire manipulation to pass the stricture. pCLE may accurately diagnose an injured nonmalignant bile duct because pCLE can provide images from 40 to 70 *μ*m below the tissue surface.

**Figure 5:**
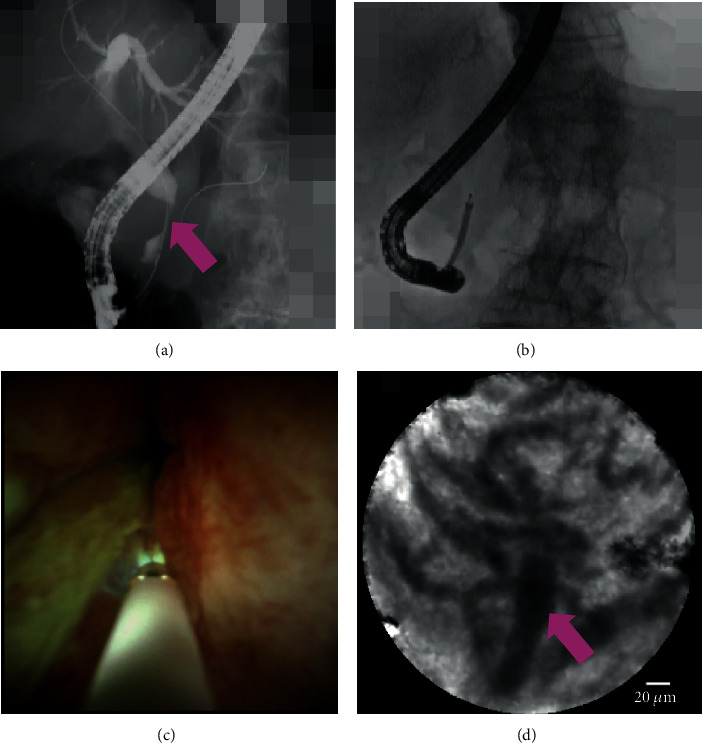
A case of autoimmune cholangitis: (a) cholangiography shows the biliary stricture (pink arrow); (b) pCLE under the direct view of POCS was performed; (c) cholangioscopy shows a reddish papillogranular surface; (d) pCLE shows a thickened reticular structure in the Paris classification.

It was thought that false-positive cases were induced by benign inflammatory conditions, resulting in a lower specificity using the Miami classification. In such cases, the Paris classification [23] is thought to be more useful. We encountered one case of adenomyosis, which was a suspected malignancy on pCLE using the Miami classification. Thickened reticular structure mentioned in the Paris classification could be seen retrospectively in this case. Moreover, among 11 cases of inflammation (chronic cholangitis, stenosis after cholecystectomy, adenomyosis, autoimmune pancreatitis, and IgG4-related sclerosing cholangitis), 6 were seen as images of inflammatory stricture in the Paris classification. Therefore, using the Paris classification may improve diagnostic ability, so we believe that the Paris classification should be selected in the future. Moreover, it is considered important to analyze the pCLE images of autoimmune (AI) cholangitis (IgG4-related sclerosing cholangitis or autoimmune pancreatitis) because it is sometimes difficult to differentiate between malignant and benign lesions in clinical practice. We had experienced 2 cases of IgG4-related sclerosing cholangitis and 4 cases of autoimmune pancreatitis shown in Table 1. We retrospectively evaluated AI cholangitis cases in this study using the Paris classification. Among 6 cases, 2 (one is autoimmune pancreatitis and the other is IgG4-related sclerosing cholangitis) were seen as images of inflammatory stricture (thickened reticular structure) in the Paris classification (Figures 2(d) and Figure 5). Other 4 cases showed features of benign lesions (reticular network of thin branching bands) in the Miami classification. Inflammatory strictures seen in the Paris classification could not be seen in all cases. This may be dependent on the degree of inflammation spread to the bile duct.

Since some studies have indicated that intravenous injection of fluorescein sodium may cause shock or arterial ischemia [24, 25], the fluorescein dripping method was applied in this study as previously described [26–30]. These studies demonstrated that local mucosal application of fluorescein showed similar pCLE findings to fluorescein via intravenous injection. Moreover, there were no adverse events using the fluorescein dripping method in these studies. Actually, some literatures reported that the diagnosis of biliary stricture utilizing the Miami classification was done with pCLE using the fluorescein dripping method and observed well [29, 30].

It is necessary to consider which timing pCLE under the direct view of POCS should be performed. Although there were good outcomes of pCLE under the direct view of POCS, it costs more expensive than usual ERCP procedures if this procedure is carried out as the initial diagnosis work-up is more expensive than that of prior ERCP. It is demanded to examine the cost-effectiveness of pCLE under the direct view of POCS. If cost-effectiveness will be proven, we should perform pCLE under the direct view of POCS as the initial diagnosis work-up.

This study has several limitations. First, this was a single-center prospective study, and the number of patients was small to show the accuracy and usefulness of pCLE under the direct view of POCS. In this study, we intended to report the feasibility of pCLE under the direct view of POCS as soon as possible. Although it showed good outcomes, more cases or a larger multicenter studies are needed. Second, tissue sampling under fluoroscopy and other cytology such as brush cytology were not performed in this study. These modalities are currently used for stricture characterization. We should have used and compared the results of these modalities with those of tissue sampling under the direct view of POCS. Third, the Miami classification was applied in this study; however, the Paris classification should be applied in the future. Furthermore, we did not compare pCLE under the direct view of POCS to pCLE under fluoroscopic guidance with ERCP. Further investigations with larger study populations are needed to make comparison with direct view and fluoroscopy guidance of pCLE.

In conclusion, pCLE under the direct view of POCS provided a highly accurate and sensitive characterization of biliary strictures and showed the potential for more diagnostic reliability and reduced delays in diagnosis.

## Figures and Tables

**Table 1:** Patient characteristics.

Patient characteristics	
Patients, *n*	30
Age, median (IQR) (years)	71 (66-74)
Sex (male/female), *n*	26/4
Preexisting condition, *n* (%)	
Cancer history	3 (10)
Cirrhosis	1 (3.3)
Congestive heart failure	3 (10)
Hypertension	9 (30)
Diabetes	5 (16.7)
Prior ERCP procedures	7 (23.3)
Prior biliary stent placement	3 (10)
Prior biliary sphincterotomy	2 (6.7)
Stricture location, *n* (%)	
Common bile duct	15 (50)
Hilar bile duct	13 (43.3)
Left main duct	2 (6.7)
Final diagnosis, *n* (%)	
Malignancy	17 (56.7)
Cholangiocarcinoma	16 (53.3)
Pancreatic cancer	1 (3.3)
Benign	13 (43.3)
Autoimmune pancreatitis	4 (13.3)
IgG4-related sclerosing cholangitis	2 (6.7)
Stenosis after cholecystectomy	2 (6.7)
Chronic cholangitis	3 (10)
Adenomyosis	1 (3.3)
Peribiliary cyst	2 (6.7)

IQR: interquartile range; ERCP: endoscopic retrograde cholangiopancreatography.

**Table 2:** Summary of primary outcomes.

Diagnosis modality (*n* = 30)	Sensitivity *n* (%) 95% CI	Specificity *n* (%) 95% CI	NPV *n* (%) 95% CI	PPV *n* (%) 95% CI	Accuracy *n* (%) 95% CI
(1) ERCP alone	15/17 (88.2) 65.7-97.9	6/13 (46.2) 23.3-70.9	6/8 (75) 40.9-95.6	15/22 (68.2) 47.3-83.6	21/30 (70) 52.1-83.3
(2) POCS	17/17 (100) 81.6-100	10/13 (76.9) 49.7-91.8	10/10 (100) 72.2-100	17/20 (85) 64.0-94.8	27/30 (90) 74.4-96.5
(3) pCLE under the direct view of POCS	16/17 (94.1) 73.0-99.7	12/13 (92.3) 66.7-99.6	12/13 (92.3) 66.7-99.6	16/17 (94.1) 73.0-99.7	28/30 (93.3) 78.7-98.8
(4) Tissue sampling under the direct view of POCS	14/17 (82.4) 59.0-93.8	13/13 (100) 77.2-100	13/16 (81.3) 57.0-93.4	14/14 (100) 78.5-100	27/30 (90) 74.4-96.5
(2) vs. (3), *P* value	0.50	0.59			>0.99
(3) vs. (4), *P* value	0.60	>0.99			>0.99

CI: confidence interval; NPV: negative predictive value; PPV: positive predictive value; ERCP: endoscopic retrograde cholangiopancreatography; pCLE: probe-based confocal laser endomicroscopy; POCS: peroral cholangioscopy.

**Table 3:** Outcomes of pCLE under the direct view of POCS.

Success of pCLE under the direct view of POCS, *n* (%)	30/34 (88.2)
Median whole procedure time, min (IQR)	70 (60.3-79.8)
Median procedural time of pCLE under the direct view of POCS, min (IQR)	42 (31.3-48)
Accurate diagnosis of the common bile duct, *n* (%)	13/15 (86.7)
Accurate diagnosis of the hilar or intrahepatic bile duct, *n* (%)	15/15 (100)
*P* value among stricture location	0.48
Adverse events, *n* (%)	
Pancreatitis	1 (3.3)
Cholangitis	2 (6.7)

pCLE: probe-based confocal laser endomicroscopy; POCS: peroral cholangioscopy; IQR: interquartile range.

## Data Availability

The patient data used to support the findings of this study are restricted by the Institutional Review Board at the Saitama Medical University International Medical Center in order to protect patient privacy. Data are available from Yuki Tanisaka (tanisaka1205@gmail.com) for researchers who meet the criteria for access to confidential data.

## Supplementary Materials

Supplementary Materials Video S1: pCLE under the direct view of POCS showed dark glandular structures with irregular margins, suggestive of malignancy.
